# X-LINKED ADRENOLEUKODYSTROPHY IN BRAZIL: A CASE SERIES

**DOI:** 10.1590/1984-0462/;2019;37;4;00015

**Published:** 2019-06-19

**Authors:** Fernanda Luiza Schumacher Furlan, Macleise Andres Lemes, Ligia Cecilia Fuverki Suguimatsu, Carolina Teixeira Furquim Pires, Mara Lucia Schmitz Ferreira Santos

**Affiliations:** a Faculdade Evangélica do Paraná, Curitiba, PR, Brazil.; b Hospital Pequeno Príncipe, Curitiba, PR, Brazil.

**Keywords:** Adrenoleukodystrophy, Peroxisomal disorders, Demyelinating diseases, Bone marrow transplantation, Aphasia, Adrenal insufficiency, Adrenoleucodistrofia, Transtornos peroxissômicos, Doenças desmielinizantes, Transplante de medula óssea, Afasia, Insuficiência adrenal

## Abstract

**Objective:**

To describe patients with different phenotypes of X-linked adrenoleukodystrophy: pre-symptomatic, cerebral demyelinating inflammatory adrenoleukodystrophy, adrenomyeloneuropathy and adrenal insufficiency only.

**Methods:**

Specific data related to epidemiology, phenotype, diagnosis and treatment of 24 patients with X-linked adrenoleukodystrophy were collected. A qualitative cross-sectional and descriptive-exploratory analysis was performed using medical records from a reference center in Neuropediatrics in Curitiba, Brazil, as well as an electronic questionnaire.

**Results:**

The majority (79%) of patients had cerebral demyelinating inflammatory adrenoleukodystrophy, presenting aphasia, hyperactivity and vision disorders as the main initial symptoms. These symptoms appeared, on average, between six and seven years of age. There was a mean delay of 11 months between the onset of symptoms/signs and the diagnosis. Patients sought diagnosis mainly with neuropediatricians, and the main requested tests were dosage of very long chain fatty acids and brain magnetic resonance.

**Conclusions:**

All phenotypes of X-linked adrenoleukodystrophy, except for myelopathy in women, were presented in the studied population, which mainly consisted of children and adolescents. Prevalent signs and symptoms registered in the literature were observed. Most of the patients with cerebral demyelinating inflammatory adrenoleukodystrophy were not diagnosed in time for hematopoietic stem cell transplantation.

## RESUMO

**Objetivo:**

Descrever pacientes com diferentes formas de adrenoleucodistrofia ligada ao X: pré-sintomática, adrenoleucodistrofia inflamatória desmielinizante cerebral, adrenomieloneuropatia e insuficiência adrenal primária.

**Métodos:**

Dados específicos relacionados a epidemiologia, fenótipo, diagnóstico e tratamento de 24 pacientes com adrenoleucodistrofia ligada ao X foram coletados. Realizou-se análise qualitativa, transversal e descritivo-exploratória, utilizando prontuários de um centro de referência em neuropediatria de Curitiba, Brasil, além de um questionário eletrônico.

**Resultados:**

A maioria (79%) dos pacientes manifestou adrenoleucodistrofia inflamatória desmielinizante cerebral, apresentando afasia, hiperatividade e distúrbios da visão como principais sintomas iniciais, que apareceram, em média, entre seis e sete anos de idade. Houve um atraso médio de 11 meses entre o início das manifestações e o diagnóstico. Os pacientes procuraram diagnóstico principalmente com neuropediatras, e os principais exames solicitados foram dosagem de ácidos graxos de cadeia muito longa e a ressonância magnética de crânio.

**Conclusões:**

Todos os fenótipos da adrenoleucodistrofia ligada ao X, exceto mielopatia em mulheres, foram apresentados na amostra estudada, composta principalmente de crianças e adolescentes. Foram observados sinais e sintomas prevalentes na literatura. A maioria dos pacientes com adrenoleucodistrofia inflamatória desmielinizante cerebral não recebeu diagnóstico em tempo hábil para a realização de transplante de medula óssea.

## INTRODUCTION

Adrenoleukodystrophy is a rare genetic disorder linked to the X chromosome (X-ALD) that affects 1:15,000-25,000 individuals worldwide, predominantly males.^1^ ^,^ ^2^ It results from mutations in the gene that encodes the peroxisomal transporter ABCD1 (adenosine triphosphate - ATP-Binding Cassette transporter subfamily D member 1), located on the long arm of the X chromosome, Xq28. Since such membrane protein is responsible for transporting very long chain fatty acids (VLCFA) into peroxisomes in order to promote their degradation by oxidation, the disorder is characterized by their accumulation in tissues and body fluids. Consequently, it leads to adrenal insufficiency and axonal demyelination.^1^ ^,^ ^3^

The exact mechanisms by which the excess of VLCFA leads to neurotoxicity are still unknown, but it has been assumed that it promotes cell membrane instability and oxidative stress.^1^ Affected individuals may present several manifestations, which vary according to the isolated, simultaneous or sequential involvement of the adrenal gland and central nervous system, and there is no correlation between the type of mutation and the phenotype presented.^2^ It has been categorized into the following forms: pre-symptomatic, cerebral demyelinating inflammatory adrenoleukodystrophy (CALD), adrenomyeloneuropathy (AMN), myelopathy among women and primary adrenal insufficiency only. The majority of cases in males evolves to adrenal insufficiency and myelopathy.^4^ When clinical manifestations of the disease are associated with alterations in the white matter, evidenced by magnetic resonance imaging (MRI) with gadolinium enhancing active demyelination, there is a strong suspicion of X-ALD. Nonetheless, the disorder is only confirmed with the levels of serum VLCFA and/or genetic testing.^4^ ^,^ ^5^

Currently, the best treatment available for the disease is hematopoietic stem cell transplantation (HSCT), which, however, only presents satisfactory results if performed at the onset of neurological symptoms, when the MRI reveals inflammatory demyelination, but when the cerebral disease burden it is still so low that the patient does not manifest clinically obvious disease. Thus, it is necessary for health professionals to be capable of identifying signs and symptoms of the disease, since it may rapidly lead to a vegetative state or death soon after the first neurological manifestations.^6^

Therefore, this research aims to raise awareness of X-ALD by describing a series of cases presenting with different forms of the disorder. Emphasis was given to the initial presentations according to the phenotype, the age in which they occurred, the age in which patients were diagnosed and the clinical outcomes in order to identify symptomatic patterns and consequently contribute to its early diagnosis and adequate treatment.

## METHOD

This study was approved by the Committee of Ethics in Research of Pequeno Príncipe Hospital, which is accredited to the National Commission of Ethics in Research of the Brazilian Ministry of Health, under the number 2.033.303 (26/04/17). It is a cross-sectional study based on:

- Analysis of clinical records of patients with X-ALD who attended a reference center in pediatric neurology in the state of Parana, Brazil.
- Application of an electronic questionnaire intended for family members of individuals with the disorder, which was created by the authors using the program Google Forms, to expand the researched population and to approach patients from other locations in the country.

In order to answer the questionnaire, they were informed about the purpose of the research and agreed to an informed consent term, confirming that they had read and understood it and accepted their participation in the research.

Specific data related to the epidemiology, disease presentation, diagnosis and treatment of 24 patients were collected. These include: gender, X-ALD phenotype, date of birth and current age / date of death, age of the onset of symptoms and description of the symptoms, age at diagnosis, professionals consulted until diagnosis, imaging and laboratory tests requested for diagnosis, therapeutics, means of financing the treatment and data related to family history.

A case of neonatal adrenoleukodystrophy, a variant of the Zellweger spectrum disorder, was excluded from the study, because of its distinct pathogenesis from X-ALD.^4^ Measures of central tendency were expressed as means and standard deviation if the data were parametric and as median with interquartile range (IQR) when non-parametric. Data analysis was performed using Windows Excel 2016.

## RESULTS

Of a total of 24 patients with X-ALD, 23 (95.8%) were male. Three variations of the disease were reported in the male subjects: CALD in 19 (79%), AMN in three (13%), and one case of primary adrenal insufficiency only. There was one case of pre-symptomatic disease, in a female individual.

**Figure 1:**
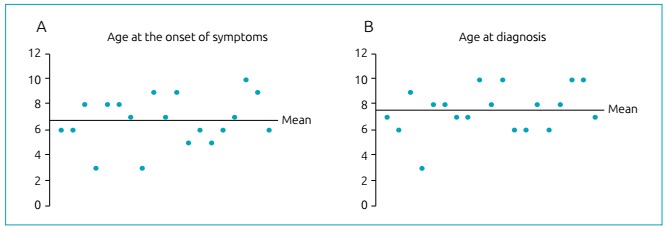
Comparison between (A) the age of the initial symptoms and (B) the age of diagnosis of cerebral demyelinating inflammatory adrenoleukodystrophy.

A greater level of detail was given on the analysis of CALD, as it was the most common phenotype among the studied group. Of the patients with CALD, four (21%) had died from complications of the disease, with nine, 12, 13 and 23 years of age. The other individuals with this phenotype had a mean age of 11.3±3.5 years old at the time of the study. Figure 1 compares the age patients with this phenotype showed the first signs and symptoms of the disease and the age they were diagnosed. The distribution of these manifestations is shown in Table 1. The mean delay between the onset of symptoms and the diagnosis of CALD was 11 months.

**Table 1:** Distribution of the initial signs and symptoms in individuals with cerebral demyelinating inflammatory adrenoleukodystrophy.

Initial signs and symptoms of CALD	Incidence (%)
Aphasia	66.7 (n=13)
Hyperactivity	57.9 (n=11)
Visual disorders	47.4 (n=9)
Poor school performance	47.4 (n=9)
Skin hyperpigmentation	42.1 (n=8)
Impaired motor coordination	42.1 (n=8)
Sphincter incontinence	36.8 (n=7)
Memory disorders	36.8 (n=7)
Aggressiveness	31.6 (n=6)
Hearing disorders	26.3 (n=5)
Decreased strength in lower limbs	26.3 (n=5)
Dysgraphia	26.3 (n=5)
Uninhibited behavior	21.0 (n=4)
Seizures	21.0 (n=4)
Difficulty in reading	21.0 (n=4)
Difficulty in ambulation	21.0 (n=4)

CALD: cerebral demyelinating inflammatory adrenoleukodystrophy.

Regarding the patients with AMN, one had died from complications of an adrenal crisis, at 24 years of age. Among the other two cases, the mean age at the time of the study was 40±4.2 years. In two patients, X-ALD started as primary adrenal insufficiency at seven and 11 years of age, manifesting with skin hyperpigmentation and vomiting, which evolved to AMN 35 and 5 years later, respectively. The mean age at which patients presented the first signs and symptoms of AMN was 29±13 years, and the age at which they were diagnosed was 31±11 years, with a mean delay of diagnosis of two years and four months. The first manifestations reported were difficulty in ambulation (all individuals), decreased strength in lower limbs (n=2) and impaired motor coordination (n=1).

The case of primary adrenal insufficiency only was of a patient whose symptoms (skin hyperpigmentation and vomiting) started at 17 years of age, in the same year of the diagnosis of X-ALD, since there was positive family history for the disease. Currently, this patient is 28 years old.

The female with the pre-symptomatic form was diagnosed with a mutation in ABCD1 at 28 years of age, after her brother was diagnosed with AMN. She is currently 48 years old and denies symptoms.

The studied population consulted on average three professionals of different specialties before receiving diagnosis of X-ALD. The most frequently referred professionals consulted until the diagnosis of CALD were pediatric neurologists (78.9%), pediatricians (47.2%), endocrinologists (36.8%), psychologists (36.8%) and neurologists (36.8%). Neurologists were also consulted by all patients with diagnosis of AMN. The other professionals mentioned by this group were orthopedists (n=2), angiologist (n=1) and physiatrist (n=1). The patient with primary adrenal insufficiency only obtained his diagnosis consulting only one pediatrician, considering that he had positive family history. Due to the same reason, the pre-symptomatic patient was diagnosed after consulting one neurologist.

Tables 2 and 3 present the examinations that were requested for investigation of CALD and AMN, respectively. For the patient with primary adrenal insufficiency only, the requested tests were skull and full spine MRI, electroencephalogram, neurological tests, and levels of sodium, potassium, renin, adrenocorticotropic hormone (ACTH), cortisol and VLCFA.

**Table 2:** Exams requested in patients with suspicion of cerebral demyelinating inflammatory adrenoleukodystrophy.

Exams requested for the investigation of CALD	Frequency (%)
Serum levels of VLCFA	100.0 (n=19)
Head MRI	94.7 (n=18)
Serum levels of cortisol	68.4 (n=13)
Head CAT scan	63.2 (n=12)
Electroencephalogram	57.9 (n=11)
Serum levels of ACTH	57.9 (n=11)
Serum levels of Na^+^	57.9 (n=11)
Serum levels of K^+^	57.9 (n=11)
Blood glucose	52.6 (n=10)
Molecular testing for X-ALD (genetic testing)	42.1 (n=8)
Neurologic performance (IQ, visuospatial ability, language, memory)	36.8 (n=7)
Urinalysis	36.8 (n=7)
Complete ophthalmologic evaluation	36.8 (n=7)

CALD: cerebral demyelinating inflammatory adrenoleukodystrophy; VLCFA: very long chain fatty acids; MRI: magnetic resonance imaging; CAT: computed tomography; ACTH: adrenocorticotropic hormone; X-ALD: adrenoleukodystrophy; IQ: intelligence quotient.

**Table 3:** Exams requested in patients with suspicion of adrenomyeloneuropathy.

Exams requested for the investigation of AMN	Frequency (%)
Serum levels of VLCFA	100.0 (n=3)
Head MRI	100.0 (n=3)
Full spine MRI	66.7 (n=2)
Abdominal CAT scan	33.3 (n=1)
Ophthalmologic evaluation	33.3 (n=1)
Neurologic performance (IQ, visuospatial ability, language, memory)	33.3 (n=1)
Molecular testing for X-ALD (genetic testing)	33.3 (n=1)
Serum levels of Na^+^	33.3 (n=1)
Serum levels of K^+^	33.3 (n=1)
Serum levels of cortisol	33.3 (n=1)
Serum levels of renin	33.3 (n=1)
Serum levels of ACTH	33.3 (n=1)
Liver function	33.3 (n=1)
Urinalysis	33.3 (n=1)
Serum levels of vitamin B	33.3 (n=1)
Lyme disease investigation	33.3 (n=1)
HIV investigation	33.3 (n=1)

AMN: adrenomyeloneuropathy; VLCFA: very long chain fatty acids; MRI: magnetic resonance imaging; CAT: computed tomography; IQ: intelligence quotient; X-ALD: adrenoleukodystrophy; ACTH: adrenocorticotropic hormone; HIV: human immunodeficiency virus.

Among the patients with CALD, follow-up was mainly with pediatric neurologists (73.7%), geneticists (26.3%) and endocrinologists (21%), and all were cared for by multidisciplinary teams. The main therapeutic resource used for CALD was diet restriction in VLCFA, in 63.2% of the patients. Similarly, the group with AMN was accompanied by neurologists (in all cases) and geneticist (in one case). These individuals also managed the disorder with diet restriction in VLCFA (n=2), as well as with Lorenzo’s Oil (n=2) and physiotherapy (n=2). The patient with primary adrenal insufficiency only consulted a pediatric neurologist and he was treated with prednisolone and Lorenzo’s Oil. The pre-symptomatic patient had no follow-up.

The main funding for treatment (58.3% of the studied group) was the Brazilian public health system (Sistema Único de Saúde - SUS).

HSCT was performed in five (20.8%) patients, four patients with CALD and the one with primary adrenal insufficiency only. Indication for HSCT was given based on radiographically evident cerebral disease. Transplant was performed when the cerebral disease was not yet symptomatic: only minimal cerebral areas affected by myelin loss and the patients were not yet manifesting any signs or symptoms of myelin loss. The patient who presented only primary adrenal insufficiency underwent HSCT due to suggestive signs of demyelination in the deep white matter adjacent to the posterior portion of the lateral ventricles and the corpus callosum and a change of signal between T6-T9 on MRI.

The main causes for hospitalization of individuals with CALD were pneumonia and seizures (47.4%), followed by upper respiratory infections (21.0%), acute gastro-enterocolitis (15.8%), febrile episodes, sinusitis and adrenal insufficiency crisis (10.5%).

The studied population had a positive family history of X-ALD in 38% of cases, half of which was represented by a brother. Only 20.8% of the cohort had been diagnosed as a result of a previous diagnosis of X-ALD in the family. On the other hand, the diagnosis of 69.6% of the studied population induced the investigation of X-ALD in other family members by genetic testing.

## DISCUSSION

The most frequent phenotypes of X-ALD are CALD and AMN, with a worldwide prevalence of 45 and 35% of cases, respectively.^1^ ^,^ ^2^ In accordance to literature, the greatest number of patients in this study presented CALD. This form affects only males, usually between five and 12 years of age, and is characterized by a rapidly progressive inflammatory demyelination of cerebral white matter, culminating in a decline in neurological function and eventually leading to total disability, followed by death at variable ages.^1^ ^,^ ^2^ ^,^ ^6^

Mahmood et al. found that the mean age of symptoms onset in patients with CALD is seven years old, the same age found in this study, and reported that 46% of patients died at an average age of 12.^7^ Our findings, on the other hand, evidenced that 21% of the patients with this phenotype had died with a mean age of 14 years old.

The first symptoms of CALD are usually disruptive behaviors and learning deficits, which may persist for months and are followed by other manifestations that indicate severity, such as aphasia, regression in writing and reading, poor school performance, impaired spatial orientation, visual disturbances, aggressive or uninhibited behavior and convulsions,^2^ ^,^ ^6^ in conformity to those ones found in the studied population. In contrast, Jiang et al. analyzed 19 cases of boys with CALD and found skin hyperpigmentation (53% of patients) as the main symptom.^3^

AMN, on the other hand, is a slowly progressive phenotype of X-ALD characterized by axonopathy with initial clinical manifestations at 20-30 years of age in men and 40-50 years of age in women, in whom symptoms are milder.^1^ ^,^ ^2^ According to Moser (2016), the mean age of occurrence of AMN is 28 years old.^8^ Similarly, we found an average age of 29 years.

The typical clinical feature of a man with AMN is difficulty in ambulation caused by stiffness and progressive weakness of lower limbs,^4^ which was presented by all individuals with AMN in this study. In addition, patients commonly present decreased vibratory sensitivity in lower limbs, sphincter dysfunction, sexual impotence and adrenal dysfunction. Considering they are non-specific symptoms, diagnosis of AMN is rarely achieved during the first three to five years of disease, unless there is family history of X-ALD,^4^ which occurred in two patients with AMN in this study.

Differently from CALD, in AMN there is no inflammatory process leading to axonal demyelination. Therefore, HSCT is not a treatment option, nor is there a curative therapy for it. Treatment is limited to symptomatic relief of adrenal or gonadal insufficiency, neuropathic pain and spasticity.^2^ Nevertheless, survival rates are estimated in decades.^6^ Adrenal insufficiency is often the first manifestation of X-ALD.^9^ There is a tendency for VLCFA to accumulate in the reticular and fasciculate zones of adrenal cortex, leading to cortisol and androgen insufficiency.^6^ ^,^ ^10^ This may occur decades before neurological symptoms, as found in this study, in which a patient with primary adrenal insufficiency developed AMN 35 years later. Not only may the insufficiency evolve to AMN, it can remain as the only manifestation of X-ALD or evolve to CALD.^2^ In 42% of CALD patients, the disease had started with skin hyperpigmentation, and, among the three patients with AMN, two had been initially diagnosed with primary adrenal insufficiency. Thus, idiopathic primary adrenal insufficiency in boys should prompt screening for X-ALD absent another identifiable cause.^10^

Regarding manifestations in women, Engelen et al. carried out a prospective cross-sectional cohort study in which female patients with X-ALD developed signs and symptoms of myelopathy and / or peripheral neuropathy over the years, and the frequency of symptomatic women sharply increased with age (from 18% of women >40 years old to 88% of women >60 years old).^11^

There is no record in the literature of the number of health professionals who are consulted until the diagnosis of X-ALD. Similar to our study, Kemp et al. reported that patients with X-ALD are usually diagnosed by a neuropediatrician, endocrinologist, or neurologist, a fact that reveals the importance of early recognition of the disease’s clinical and radiological features by these professionals.^6^

MRI always shows abnormalities in symptomatic patients and it is usually the first clue to diagnosis. In approximately 85% of affected individuals, MRI shows a characteristic T2-weighted hypersignal symmetrical pattern in parieto-occipital region with contrast enhancement at the margins.^11^ ^,^ ^12^ In T1-weighted MRI, contrast enhancement around demyelinating lesions can be observed, reflecting brain barrier dysfunction and, therefore, a higher probability of brain disease progression. Global brain atrophy is a late event and indicates a debilitating disease.^13^ Such alterations are classified using a system that identifies the severity of white matter lesions on a scale from 0 (normal) to 34 (severely abnormal), also known as Loes Scale.^4^ ^,^ ^5^ ^,^ ^14^

The most important laboratory test is the plasma VLCFA level, which was performed by all patients in our study. It is usually enough to confirm the diagnosis in affected male patients, since there is a high concentration of VLCFA in males with X-ALD in all ages. However, when X-ALD is suspected in a female patient, it is also necessary to request a genetic test, as up to 15% of female patients with X-ALD have normal values of VLCFA.^12^ ^,^ ^15^

A confirmation of X-ALD diagnosis by the analysis of mutations in ABCD1 is especially advisable in patients with atypical symptoms or when HSCT is considered.^11^ In addition to identifying female carriers, as described with the pre-symptomatic patient in this study, the analysis of mutations in ABCD1 in family members is essential.^2^

The importance of early identification of individuals with X-ALD in order to promote a better prognosis has caught the attention of public health authorities in some countries, including the United States and the Netherlands, to promote newborn screening. It is a breakthrough, as it can ensure the diagnosis of in optimal time to promote an adequate management of the patient.^4^ ^,^ ^8^ ^,^ ^16^ ^,^ ^17^ ^,^ ^18^ Therefore, its implementation in Brazil would avoid the distress of families in search of a diagnosis for their sick child. In addition, it would facilitate medical conduct. Challenges to be addressed by neonatal X-ALD screening include the difficulty in predicting the patient’s clinical manifestations, as there is a poor correlation between the genotype found and the manifested phenotype, which may increase the risk of unnecessary treatments.^16^ Furthermore, introducing an X-ALD screening programme requires the participation of geneticists, genetic counsellors and policy makers to evaluate its feasibility, protocols, risks, and benefits.^17^

The most mentioned therapeutic resource in the present study was dietary restriction of VLCFA. However, as Percy and Rutledge indicated, this approach by itself does not reduce the concentration of VLCFA due to its endogenous synthesis. Therefore, the combination of Lorenzo’s Oil (4:1 mixture of oleic acid and erucic acid) with the diet is particularly useful, since it can reduce internal VLCFA.^19^ Other authors found that the oil is not capable of altering the progression of brain disease in affected individuals, so it is not indicated after brain involvement.^6^ Thus, it is mainly used in AMN,^5^ as occurred in this study.

When the neuro-inflammatory process is detected at an early stage, it can be interrupted by HSCT or gene therapy.^1^ ^,^ ^2^ In CALD, the HSCT mechanism of action remains unclear, but it is believed that donor microglial cells provide support to the receptor oligodendrocyte in the combat against the neuro-inflammatory process.^19^ Yet, it was performed in less than a quarter of patients in this study. Shapiro et al. studied 18 boys who received HSCT at an early-stage of X-ALD and described remarkable benefits: 44% of patients returned to school without additional support, motor function improved in 56%, verbal intelligence was stabilized in 61%, and performance tests improved or stabilized in 39%.^20^

The timing between first manifestations and diagnosis of CALD in our study should have been much faster, according to the literature, since there may be total disability within six to 24 months of disease progression, preventing HSCT and consequently the interruption of the inflammatory demyelination.^1^

Due to the systemic manifestations, patients have a multidisciplinary follow-up. Moreover, treatment requires a wide range of medications and interventions, which include periods of hospitalization.^2^ The incidence of seizures, in this study, was higher than described in the literature (20% in affected boys).^21^ This represents high cost to the public health system,^22^ a fact that is evidenced in this study, since most patients had the public health system as the main means of treatment. Consequently, SUS’ authorities included X-ALD among 12 rare diseases that will receive clinical protocols in 2018, aiming to reduce mortality and improve the quality of life of patients and their families.^22^

Implementation of a newborn screening program in Brazil and genetic testing of family members would greatly improve the management of individuals with ABCD1 mutations. In addition, extensive screening of at-risk family members should follow any identification of patient or carrier. Furthermore, it would be relevant in the field of public health to carry out a comparative study on the costs of treatment with HSCT for all patients who are suitable for so and the costs of alternative therapies and consequent hospitalizations.

As potential limitations of this study, there was insufficient detailed clinical data of some patients, since part of the research was based on an electronic questionnaire, answered by patients and their relatives. Also, there was lack of registration of the Loes Score in medical records, which prevented a correlation of the score to disease severity. In addition, it would be interesting, for future studies, to conduct a longer follow-up of patients.

In summary, all phenotypes of X-ALD, except for myelopathy in women, were presented in the studied population, which mainly consisted of children and adolescents. Prevalent signs and symptoms registered in the literature were observed. Nonetheless, most of the patients with CALD were not diagnosed in time for HSCT.
